# Associations between branched chain amino acid intake and biomarkers of adiposity and cardiometabolic health independent of genetic factors: A twin study^☆^

**DOI:** 10.1016/j.ijcard.2016.08.307

**Published:** 2016-11-15

**Authors:** Amy Jennings, Alex MacGregor, Tess Pallister, Tim Spector, Aedín Cassidy

**Affiliations:** a Department of Nutrition and Preventive Medicine, Norwich Medical School, University of East Anglia, Norwich, UK; b Department of Twin Research and Genetic Epidemiology, Kings College London, London, UK

**Keywords:** BCAA, branched chain amino acids, DBP, diastolic blood pressure, DZ, dizygotic, FFQ, food frequency questionnaire, HDL-C, high density lipoprotein cholesterol, hs-CRP, high sensitivity C-reactive protein, MZ, monozygotic, SBP, systolic blood pressure, T2DM, type 2 diabetes, SBP, systolic blood pressure, WHtR, waist to height ratio, Cardiometabolic, Diet, Amino acids

## Abstract

**Background:**

Conflicting data exist on the impact of dietary and circulating levels of branched chain amino acids (BCAA) on cardiometabolic health and it is unclear to what extent these relations are mediated by genetics.

**Methods:**

In a cross-sectional study of 1997 female twins we examined associations between BCAA intake, measured using food frequency-questionnaires, and a range of markers of cardiometabolic health, including DXA-measured body fat, blood pressure, HOMA-IR, high-sensitivity C-reactive protein (hs-CRP) and lipids. We also measured plasma concentrations of BCAA and known metabolites of amino acid metabolism using untargeted mass spectrometry. Using a within-twin design, multivariable analyses were used to compare the associations between BCAA intake and endpoints of cardiometabolic health, independently of genetic confounding.

**Results:**

Higher BCAA intake was significantly associated with lower HOMA-IR (− 0.1, *P*-trend 0.02), insulin (− 0.5 μU/mL, *P*-trend 0.03), hs-CRP − 0.3 mg/L, *P*-trend 0.01), systolic blood pressure (− 2.3 mmHg, *P*-trend 0.01) and waist-to-height ratio (− 0.01, *P*-trend 0.04), comparing extreme quintiles of intake. These associations persisted in within-pair analysis for monozygotic twins for insulin resistance (*P* < 0.01), inflammation (*P* = 0.03), and blood pressure (*P* = 0.04) suggesting independence from genetic confounding. There was no association between BCAA intake and plasma concentrations, although two metabolites previously associated with obesity were inversely associated with BCAA intake (alpha-hydroxyisovalerate and trans-4-hydroxyproline).

**Conclusions:**

Higher intakes of BCAA were associated, independently of genetics, with lower insulin resistance, inflammation, blood pressure and adiposity-related metabolites. The BCAA intake associated with our findings is easily achievable in the habitual diet.

## 1 Introduction

Conflicting results have been reported on the relationship between branched chain amino acids (BCAA), which includes the essential amino acids leucine, isoleucine and valine, and cardiometabolic health. Higher fasting plasma concentrations of BCAA have been associated with an increased risk of developing type 2 diabetes and insulin resistance in both rodent models and humans [1] with several longitudinal studies observing that higher circulating levels of BCAA are associated with a threefold higher risk of type 2 diabetes and 1.3-fold higher risk of metabolic syndrome [2], [3]. Together these data have led to the suggestion that increased blood levels are predictive of future insulin resistance or type 2 diabetes.

In sharp contrast, BCAA supplementation and diets rich in BCAA intake have been shown to be beneficial for metabolic health (regulation of body weight and glucose homeostasis), especially in rodent models. Specifically, increasing leucine intake in mice fed a high-fat diet resulted in a 32% reduction in weight gain, a 25% decrease in adiposity and a 53% decrease in LDL-cholesterol concentrations independent of adiposity [4]. Improved body weight, glucose tolerance and insulin sensitivity were also observed in mice fed a BCCA-rich protein source (whey protein isolate) [5]. A previous small-scale study in 296 males reported higher BCAA intake (0.03 g/kg body weight) was associated with reduced weight (13.8 kg), total body fat (4.5%), insulin concentrations (2.1 μU/mL), diastolic blood pressure (3.4 mmHg) and a 47% lower prevalence of metabolic syndrome [6].

One proposed explanation for these conflicting findings is that circulating BCAA are a biomarker of impaired insulin action, rather than a causative factor of insulin resistance, via activation of mammalian target of rapamycin complex 1 signalling or accumulation of mitotoxic metabolites that subsequently cause mitochondrial dysfunction and apoptosis associated with type 2 diabetes [1]. Direct and indirect mechanisms for the positive effects of BCAA intake have been suggested including direct effects on hypothalamus derived processes associated with satiety and body weight and enhanced insulin-stimulated AKT phosphorylation and glucose uptake related to improved insulin sensitivity and glucose metabolism [7], [8].

To our knowledge there are no comprehensive population based studies examining the associations between BCAA intake and parameters of adiposity and cardiometabolic health and no studies utilizing a twin model to explore the relative contributions of diet and genetics and reduce confounding. We firstly examined the associations between intake and circulating levels of BCAA and then investigated the relationship between intakes and a range of parameters of metabolic health, including overweight, insulin resistance, inflammation and blood pressure, using this unique approach. On the basis of previous research, we expected that participants with higher BCAA intake would have lower adiposity and healthier parameters of cardiometabolic health; additionally we hypothesized that the associations would remain significant within monozygotic (MZ) and dizygotic (DZ) twin-pairs, suggesting a lack of genetic confounding.

## 2 Methods

### 2.1 Study population

Participants included in these analyses were twins enrolled in the TwinUK cohort [9]. The participants were not selected for particular diseases or traits and were unaware of the specific hypotheses being tested. The cohort has previously shown to be representative of the general UK population in terms of disease- and lifestyle-related characteristics, including diet [10], [11]. In this study we included female twins, aged 18–76 y, who had completed a food frequency questionnaire (FFQ) and attended a clinical assessment for cardiometabolic risk factors between 1996 and 2000. In total, 4181 unique participants completed a FFQ, of which 22% (*n* = 919) were either incomplete (> 10 food items were left blank) or participants reported implausible energy intake (defined as the ratio of energy intake to estimated basal metabolic rate falling ≥ 2SD from the population mean). Of the 3262 participants who completed a valid FFQ, 61% (*n* = 1997) attended for a relevant clinical assessment. Although 3299 participants had metabolomics data, clinical data were unavailable for 2360 of these participants, leaving us with 28% (*n* = 939) to include in the current analyses. Although the sample size was fixed before the start of the study a formal power calculation revealed that a moderate association with blood pressure of 2 mmHg (α = 0.05, 95% power) would require 913 participants in these multivariate analyses. The study was approved by the St. Thomas' Hospital Research Ethics committee and all subjects provided informed written consent.

### 2.2 Assessment of cardiometabolic biomarkers

Anthropometric measurements were made in light clothing, height was measured using a wall-mounted stadiometer to the nearest 0.5 cm, weight (light clothing only) was measured with digital scales to the nearest 0.1 kg and waist circumference (cm) was measured at the level midway between the lower rib margin and the iliac crest. These data were used to derive BMI (kg/m^2^) and waist to height ratio (WHtR). Body fat was measured by dual-energy X-ray absorptiometry using standard protocols (QDR-2000 W, Hologic, Massachusetts, USA) and percentage body fat was calculated as (body fat (kg)/total body mass (kg)) × 100. Overweight was defined if BMI was > 25 kg/m^2^ and abdominal obesity if WHtR ratio was > 0.5. Data on WHtR and body fat was available for 1828 (92%) and 1792 (90%) of the study population, respectively.

Serum samples obtained from venous blood samples were collected between 8 and 10 am after an overnight fast. Insulin was measured by immunoassay (Abbott Laboratories Ltd., Maidenhead, UK), and glucose using an enzymatic colorimetric assay (Ektachem 700 multichannel analyzer, Johnson and Johnson Clinical Diagnostic Systems, Amersham, UK). HOMA-IR was calculated according to the formula: fasting insulin × fasting glucose / 22.5. Insulin resistance was defined if HOMA-IR was ≥ 2.5 arbitrary units. We excluded participants with insulin values above clinically realistic values (400 pmol/L) or with hyperglycaemia or potential type 2 diabetes (defined as glucose values above 7 mmol/L). High sensitivity C-reactive protein (hs-CRP) was measured by a highly sensitive automated micro particle capture enzyme immunoassay (Abbott Laboratories). Inter- and intra-assay CV were < 10% throughout the range for all biomarkers. Systemic inflammation was defined as an hs-CRP value ≥ 3 mg/L. Data on hs-CRP were available for 1432 of the 1997 participants who were assessed for insulin resistance.

Peripheral systolic (SBP) and diastolic (DBP) blood pressure were measured by a trained nurse or research assistant using an automated cuff sphygmomanometer (OMRON HEM713C, Tokyo, Japan) with the participant in the seated position for at least three minutes prior to taking three measurements. Hypertension was defined as a SBP above 140 mmHg and/or diastolic blood pressure above 90 mmHg and/or use of anti-hypertensive drugs. Blood pressure data was available for 1952 (98%) of the participants included in these analyses.

Levels of all lipids were measured by using a Cobas Fara machine (Roche Diagnostics). High density lipoprotein cholesterol (HDL-C) and triglycerides (TG) were determined by a colorimetric enzymatic method. HDL-C was determined after precipitation of larger particles (chylomicron,VLDL, and LDL) by magnesium and dextran sulfate. Lipid levels were expressed as *log*TG/HDL-C as this measure has shown to be strongly correlated with cardiometabolic risk [12]. Dyslipidemia was defined as HDL-C ≤ 1.3 mmol/L and TG ≥ 1.7 mmol/L or use of cholesterol lowering drugs. Data were available for 1845 (92%) of these participants.

According to the NCEP ATP III criteria, metabolic syndrome was defined in the presence of three or more of the following: glucose ≥ 6.1 mmol/L, TG ≥ 1.7 mmol/L, HDL-C ≤ 1.3 mmol/L, waist circumference ≥ 88 cm or elevated blood pressure (SBP ≥ 130 and/or DBP ≥ 85 and/or antihypertensive drug treatment) [13]. Complete data on metabolic syndrome was available for 1663 (83%) participants.

### 2.3 Metabolomic analyses

In a sample of 3299 participants, non-targeted metabolite detection and quantification were conducted (Metabolon, Inc., Durham, NC) on fasting plasma samples using ultra-high performance liquid chromatography and gas chromatography mass spectrometry platforms, as described previously [14]. Of the metabolites identified, 75 were determined to be representative of amino acid metabolism (listed in Supplemental Table 1) and were included in these analyses [15].

### 2.4 Assessment of dietary intakes

Participants completed a 131-item validated FFQ [16], [17]. Nutrient values were assigned to each item in the FFQ or for mixed dishes a value for each ingredient, using data from UK food composition tables [18]. Amino acid content was derived predominantly using UK food composition data with additional data from the US Department of Agriculture if UK data was not available [19], [20]. If the sum of the individual amino acids and the values for protein from the latest UK food composition tables differed for a specific food item the amino acid values were rescaled to match the most recent protein data [19]. Nutrient intakes were calculated as the frequency of each food multiplied by the nutrient content of the food for the appropriate portion size [21]. We set quantitative limits based on confidence intervals to define dietary under-reporters using values of reported energy intake and total predicted energy expenditure [22]. These calculations accounted for the within-subject variation inherent in the methods used to assess energy intake and expenditure [23]. It has been shown that excluding potential under-reporters can introduce considerable bias into a sample and therefore we considered the ratio of energy intake to estimated energy expenditure as a covariate in all multivariable models [24].

### 2.5 Assessment of covariates

Information on smoking, medication use and menopausal status was obtained by standardized nurse-administered questionnaire. Physical activity was classified as inactive, moderate, and active during work, home and leisure time using a questionnaire strongly correlated with a more in-depth assessment of activity levels in this cohort [25]. The estimated active time per week for these physical activity levels is: inactive 16 min; light activity 36 min; moderate activity 102 min; and heavy activity 199 min [25]. Zygosity was ascertained by questionnaire and confirmed via subsequent genotyping as part of genome-wide association studies (PE Applied Biosystems, Foster City, California).

### 2.6 Statistical analysis

Firstly, we used all the participants, treating twins as individuals (individual level analysis) while accounting for twin-pair clustering. Data were available for 1997 (61%) of the 3262 participants who completed a valid FFQ (960 twin pairs and 77 individuals). Participants were ranked into quintiles of BCAA intake expressed as a percentage of total protein and associations with cardiometabolic parameters were determined using ANCOVA. Prevalence ratios for insulin resistance, metabolic syndrome, inflammation and overweight were estimated by quintile of BCAA intake using Poisson regression.

In further analyses we excluded singletons from the analyses (*n* = 77) and examined associations between BCAA intake, in quintiles, and cardiometabolic variables in models that included the twin-pair mean for BCAA intake as follows:

$$E\left(Y_{\mathrm{ij}}\right)=\beta_{0}+\beta_{i}X_{\mathrm{ij}}+\beta_{t}{\overset{-}{X}}_{i}$$

where *Y*_*ij*_ and *X*_*ij*_ represent the cardiometabolic (Υ) and BCAA intake variable (X) of twin *j* from family *i* and $\overset{-}{X}$ is the twin-pair mean [26]. *β*_*t*_ can be interpreted as the result of a 1-quintile increase in the pair-average of BCAA intake on cardiometabolic health with the individual intake held fixed (between-pair association). *β*_*i*_ is the result of a one-quintile increase in the individuals BCAA intake on cardiometabolic parameters with the pair average held fixed (within-pair association). Within-pair associations are inherently controlled for shared environmental factors and, in MZ pairs, genetic confounding.

All models were adjusted for age (years), current smoking (yes or no), physical activity (inactive, moderately active, active), BMI (kg/m^2^), use of hormone replacement therapy (yes or no), use of diabetes or cholesterol lowering drugs (yes or no), use of vitamin supplements (yes or no), menopausal status (pre- or post-menopausal), under-reporting (yes or no) and intakes of energy (kcal), carbohydrate (g), saturated fat (g), wholegrains (g), alcohol (g), and protein (g). Natural-log-transformed values were used as the distribution of cardiometabolic outcome measures were skewed, with the exception of lipid levels expressed as *log*TG/HDL-C. Values in the text are means or geometric means (95%CI) except for values from within-pair analyses which are the geometric means expressed as a percentage of the non-transformed values, calculated as:

$$\left[{(\exp}^{\beta }\right)-1]*100$$

Linear regression was used in multivariable analyses to determine associations between BCAA intake and the 75 metabolites associated with amino acid metabolism [15]. A separate random intercept model was performed for each metabolite with age, BMI, batch effects, family relatedness, smoking status and energy intake included as covariates:

$$\Upsilon_{i}=\beta_{0}+\beta_{i}{Χ}_{\mathrm{ij}}+\gamma_{i}{\mathrm{age}}_{\mathrm{ij}}+\delta_{i}{\mathrm{BMI}}_{\mathrm{ij}}+\zeta_{j}+\varepsilon_{\mathrm{ij}}$$

where Y_*i*_ is the metabolite and X_*ij*_ is the BCAA intake of twin *j* from pair *i*, and ζ_*j*_ is the family-specific error component that captures the unobserved heterogeneity or family characteristics.

*P*-values < 0.05 were considered statistically significant for all analyses except for the metabolomic models where we accounted for multiple testing using Bonferroni correction, giving a significance threshold of 6.67 × 10^− 4^. Statistical analyses were performed with Stata statistical software version 11 (StataCorp, Texas, USA).

## 3 Results

The characteristics, cardiometabolic risk factors and dietary intakes of the study participants are shown in Table 1. BCAAs contributed to 18.1% (SD 0.4 range 16.5 to 20.3) of protein intake with leucine (43.7% SD 0.4 range 41.7 to 46.3) contributing more than isoleucine (25.9% SD 0.4 range 24.2 to 27.0) and valine (30.4% SD 0.4 range 29.3 to 32.7) to total BCAA intake (data not shown). Compared to the participants included in the hs-CRP analyses (*n* = 1432) those excluded for having missing data (*n* = 565) were significantly younger (45.2 y vs. 47.9 y, *t* = − 4.49 *P* < 0.01) and therefore less likely to be post-menopausal (χ^2^ = 18.2 *P* < 0.01) or to take HRT (χ^2^ = 8.1 *P* < 0.01). These participants also had higher saturated fat intakes (27.5 g vs. 26.0 g, *t* = 2.9 *P* < 0.01), were more likely to smoke (χ^2^ = 5.5 *P* = 0.02) and less likely to take vitamin supplements (χ^2^ = 6.7 *P* = 0.01). There were no significant differences between those participants included (*n* = 1972) or excluded (*n* = 205) from the body fat analyses (data not shown).

There was a difference in BCAA intake between extreme quintiles of 1.3 g or 1.2% of protein (Table 2). In multivariable analyses a higher BCAA intake was associated with a lower WHtR (Q5-Q1 = − 0.01, *P*-trend 0.04) and there was a trend towards a lower body weight although this finding did not reach levels of statistical significance (Q5-Q1 = 0.7 kg, *P*-trend 0.05). Higher BCAA intake was also associated with significantly lower insulin resistance as indicated by lower HOMA-IR (Q5-Q1 = − 0.1, *P*-trend 0.02) and insulin concentrations (Q5-Q1 = − 0.5 μU/mL, *P*-trend 0.03) in addition to lower hs-CRP levels (Q5-Q1 = − 0.3 mg/L, *P*-trend 0.01) and lower SBP (Q5-Q1 = − 2.3 mmHg, *P*-trend 0.01) comparing those in the highest and lowest quintiles of intake.

**Fig. 1:**
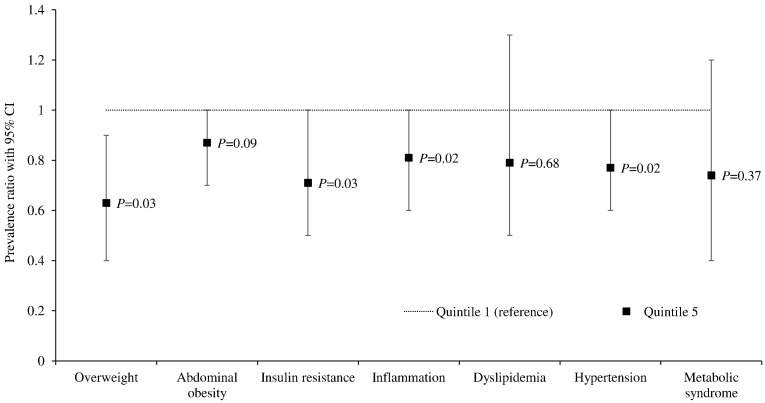
Prevalence ratios for obesity and cardiometabolic risk factors in the highest vs. lowest quintile of BCAA intake in females aged 18–76 y^1^ ^1^Values are adjusted prevalence ratios (95% CI), *n* = 1997. P = P-trend across quintiles of intake calculated using ANCOVA. Prevalence ratios are adjusted for age, smoking, physical activity, BMI, HRT, menopausal status, use of diabetes or cholesterol lowering drugs, vitamin supplements, under-reporting, and intakes of energy, carbohydrate, saturated fat, wholegrains, alcohol and protein. Overweight and abdominal obesity are not adjusted for BMI. Insulin resistance = HOMA-IR ≥ 2.5; systemic inflammation = hs-CRP ≥ 3 mg/L, dyslipidemia = HDL-C ≤ 1.3 mmol/L and TG ≥ 1.7 mmol/L or use of cholesterol lowering drugs; overweight = BMI ≥ 25 kg/m^2^; abdominal obesity =  waist to height ratio ≥ 0.5; hypertension = systolic blood pressure ≥ 140 mmHg and/or diastolic blood pressure ≥ 90 mmHg and/or anti-hypertensive drug treatment; metabolic syndrome = three of the following risk factors, glucose ≥ 6.1 mmol/L, TG ≥ 1.7 mmol/L, HDL-C ≤ 1.3 mmol/L, waist circumference ≥ 88 cm or elevated blood pressure (systolic ≥ 130 and/or diastolic ≥ 85 and/or antihypertensive drug treatment). Full data is presented in Supplemental Table 1. Subset analysis: inflammation = 1432; dyslipidemia = 1845; abdominal obesity = 1828; hypertension = 1952; metabolic syndrome = 1663.

In our cardiometabolic risk factor analyses participants with higher BCAA intake had a lower prevalence of overweight, insulin resistance, systemic inflammation, and hypertension (Fig. 1 and Supplemental Table 2). There was no association between BCAA intake and prevalence of metabolic syndrome, dyslipidemia or abdominal obesity.

In analyses controlling for genetic and shared environmental confounders in twin-pairs, significant within-pair associations between BCAA intake and markers of insulin resistance, inflammation and blood pressure were observed (Table 3). Specifically, a one-quintile higher intake of BCAA was associated with lower HOMA-IR (2.1% 95% CI − 3.9, − 0.2 *P* = 0.03), insulin (2.0%, 95% CI − 3.8, − 0.2 *P* = 0.03) and hs-CRP (7.1% 95% CI − 11.7, − 2.3 *P* < 0.01). After stratifying by zygosity we observed a greater magnitude of association for MZ than DZ twins, suggesting that the associations were free of genetic confounding (HOMA-IR − 5.0% 95% CI − 8.1, − 1.9 *P* < 0.01; insulin − 4.8% 95% CI − 7.7, − 1.8 *P* < 0.01; hs-CRP − 9.7% 95% CI − 17.7, − 1.0 *P* = 0.03; diastolic blood pressure − 1.2% 95% CI − 2.3, − 0.1, *P* = 0.04). No within-pair associations were observed for weight status variables or cholesterol levels. We observed no significant associations in our between-twin analyses when all twins were considered together or when stratified by zygosity (data not shown).

BCAA intake was unrelated to circulating levels in the 3299 participants for whom data were available (leucine β = 0.005 95% CI − 0.07, 0.05 *P* = 0.91, valine β = − 0.01 95% CI − 0.06, 0.09 *P* = 0.76 and isoleucine β = − 0.04 95% CI − 0.11, 0.04 *P* = 0.35, Supplemental Table 1). After Bonferroni correction, two of the 75 metabolites identified to be representative of amino acid metabolism were significantly inversely associated with BCAA intake, alpha-hydroxyisovalerate (β = − 0.15 95% CI − 0.23, − 0.07 *P* = 2.99 × 10^− 4^) and trans-4-hydroxyproline (β = − 0.20 95% CI − 0.28, − 0.12 *P* = 1.48 × 10^− 6^).

## 4 Discussion

In the current study, using a co-twin design, significant inverse associations were observed in female twins between dietary intakes of BCAA and measures of insulin resistance, inflammation and blood pressure, associations that were independent of a wide range of known cardiometabolic risk factors including smoking, physical activity, BMI and medication use. To our knowledge, this was the first study to examine these associations within a twin population which provided us with a unique opportunity to control for genetic confounding. With stronger findings observed in MZ compared to DZ twins, these results suggest that shared environmental and genetic factors do not confound the reported associations. Additionally, in metabolomic analyses, we identified two metabolites that were inversely associated with BCAA intake; alpha-hydroxyisovalerate and trans-4-hydroxyproline. Interestingly higher circulating levels of alpha-hydroxyisovalerate have previously been associated with greater adiposity and blood pressure and circulating levels of trans-4-hydroxyproline have shown to be elevated in patients with dietary-induced non-alcoholic fatty liver disease, a cardio-metabolic risk factor for the development of diabetes [27], [28], [29]. We found no association between BCAA intake and circulating levels confirming previous findings in children and adding evidence to support the theory that plasma BCAA levels are not a direct reflection of diet-derived intakes [30].

Genetic factors are strong determinants of dietary habits and cardiometabolic health, with estimates of heritability reported between 43 and 57% [11], [31]. Our within-twin analysis confirmed our individual-level findings for insulin resistance and inflammation but not those for measures of weight status which may indicate that previously reported associations between BCAA intake and weight status are influenced by genetics or other lifestyle factors not considered [6], [32]. We found no associations between BCAA intake and lipid levels or prevalence of metabolic syndrome in our individual- or twin-level analyses. It is noteworthy that the prevalence of clinical dyslipidemia and metabolic syndrome were very low in this apparently healthy cohort (9%).

Our results are consistent with a short-term (2 month) intervention trial which observed improvements in insulin resistance and β-cell function in men with chronic liver disease but no significant effect on body composition following supplementation with 3.2 g of BCAA (0.8 g valine, 1.6 g leucine and 0.8 g isoleucine) [33]. Our findings however, only partly support those of a longer-term supplementation trial (7.1 g leucine for 6 months) in which both glycemic control and body composition were not changed in diabetic males [34]. Our novel data also provide mechanistic support for the reported association between higher BCAA intake (0.9% of protein) and a 43% decreased risk of diabetes in Japanese women [35]. Furthermore, our findings are plausible given the findings from mechanistic studies; Macotela *et al*. recently reported, using rodent models, that doubling dietary leucine for eight weeks reversed many of the metabolic abnormalities associated with high fat diet-induced obesity, improving glucose tolerance, insulin signaling and inflammation in the adipose tissue. Interestingly, they found that serum cholesterol levels were not changed by leucine administration [36]. Our associations between BCAA intake and cardiometabolic health were independent of total protein intake and it is noteworthy that there were no associations between total protein intake and any of the cardiometabolic factors examined in these analysis (data not shown). This suggests an independent effect of BCAA rather than BCAA acting as a marker of total protein intake.

Our study results have potential clinical significance. Previous studies have shown that reduced insulin resistance and inflammation are associated with lower rates of obesity, type 2 diabetes and cardiovascular disease [37]. In our study, the highest quintile of BCAA intake compared with the lowest quintile was associated with 19–37% lower insulin resistance, inflammation, overweight and hypertension. The presence of insulin resistance (HOMA-IR > 2) was previously related to an increased odds ratio of 1.54 (95% CI 0.91–2.62) for coronary artery disease and elevated CRP (hs-CRP > 3 mg/L) was associated with odds ratios of 1.46 (95% CI 1.05–2.04) for cardiovascular disease and 3.12 (95% CI 1.77–5.48) for type 2 diabetes [38], [39], [40]. Although the associations we observed in absolute terms may be considered modest it has previously been shown that small changes in risk factors such as cholesterol, blood pressure and obesity are associated with potentially clinically relevant changes to cardiovascular risk [41].

To place our findings into context we examined the standardized beta coefficients for the HOMA-IR model which allowed us to compare the magnitude of associations for the various predictors within our model. The size of the standardized coefficient for BCAA intake (− 0.05 95% CI − 0.09, − 0.01) was more than half that of smoking, a major risk factors for poor cardiometabolic status, and 1.6 and 7 times greater than alcohol intake and age, respectively. The major dietary sources of BCAA in this cohort were milk (16.3% of intake), red meat (11.1% of intake), poultry (8.8% of intake) and high fat dairy products (5.8% of intake). The difference in intakes of BCAA between extreme quintiles of intake in the current study was 1.3 g/day, to incorporate these levels of BCAA into the diet people would need to consume either a glass of milk (185 g), one small piece of cheddar cheese (20 g), one small portion of cashew nuts (35 g), or approximately one quarter of a beef or salmon steak (28 g). Although this establishes that the relationships we have reported are related to dietary achievable intakes of BCAA further research is required before recommendations on BCAA intakes in relation to metabolic health can be made.

Strengths of the current study include the large sample of well characterised participants and the use of the co-twin model which allowed us to examine these associations independently of genetic confounding. It was notable that we excluded participants with high glucose levels (> 7 mmol/L) and there were low proportion of participants with metabolic syndrome and dyslipidemia (< 10%), as it is plausible that we would have observed greater associations if participants with impaired metabolic function were included. A further strength was the range of robust measures of cardiometabolic status, which included dual-energy X-ray absorptiometry measured body composition and HOMA-IR which is a reliable measure of *in vivo* insulin resistance and correlates well with scores obtained from the hyperinsulinemic-euglycemic clamp technique [42]. There were also limitations. There is a lack of evidence to show the FFQ used in the current study is able to accurately quantify BCAA intake although it has the ability to reflect habitual dietary intake and rank participants according to amino acid and protein intake [11], [43], [44]. We cannot infer causality from this cross-sectional study and therefore intervention studies are needed. Furthermore, although we adjusted for a range of confounders (such as age, smoking, physical activity, BMI, medication use, and intake of other nutrients associated with cardiometabolic health), there was still the possibility of residual or unmeasured confounding from additional unmeasured factors. Although we made a number of hypothesis-driven comparisons in this study we believe they were justified given the novel and exploratory nature of the analyses.

In conclusion, these novel data suggest that habitual intake of BCAA was inversely associated with parameters of insulin resistance, inflammation and blood pressure independent of shared genetic and common environmental factors. In addition, from our metabolomic dataset we identified two biomarkers of BCAA intake that have previously been associated with adiposity, although these findings need to be replicated in other data sets. Our results have clinical and public health relevance as our findings were related to dietary achievable intakes. These findings support the hypothesis that BCAA have cardio protective effects through improvements in insulin resistance, inflammation and blood pressure and highlight the need for more intervention trials examining dietary attainable levels of BCAA and metabolic health.

## Conflict of interest

The authors report no relationships that could be construed as a conflict of interest.

## Figures and Tables

**Table 1:** Characteristics of females aged 18–76 y^a^.

	Value
*Characteristics*	
Age (y)	41.7 ± 12.1 (39, 56)
Zygosity (n % monozygotic)	23.2 (464)
BMI (kg/m)^2^	25.2 ± 4.5 (22.1, 27.3)
Current smoker (yes; n %)	17.5 (350)
Physically active (yes; n %)	26.7 (533)
Hormone replacement therapy use (yes; n %)	17.5 (349)
Post-menopausal (yes; n %)	44.7 (893)
Diabetes or cholesterol lowering drug use (yes; n %)	1.7 (34)
Vitamin supplement use (yes; n %)	53.3 (1064)

*Adiposity and cardiometabolic markers*	
Body fat (% total body mass)	35.0 ± 7.5 (29.8, 40.2)
Weight (kg)	66.5 ± 12.1 (57.9, 72.5)
Overweight (BMI ≥ 25)	12.8 (255)
Waist to height ratio	0.5 ± 0.1 (0.4, 0.5)
Abdominal obesity (waist to height ratio ≥ 0.5)	39.1 (715)
HOMA-IR	1.6 ± 1.6 (0.8, 1.7)
Fasting insulin (μU/mL)	7.8 ± 6.8 (4.2, 8.6)
Fasting glucose (mmol/L)	4.5 ± 0.5 (4.2, 4.8)
Insulin resistance (HOMA-IR ≥ 2.5)	11.9 (238)
hs-CRP^b^ (mg/L)	2.5 ± 2.4 (0.7, 3.6)
Systemic inflammation (hs-CRP ≥ 3)	31.4 (450)
Lipids (*log*TG:HDL-C)	0.07 ± 0.34 (− 0.16, 0.25)
Dyslipidemia (HDL-C ≤ 1.3 and TG ≥ 1.7 or statin use)	7.9 (145)
Systolic blood pressure (mm Hg)	121 ± 16.6 (109, 131)
Diastolic blood pressure (mm Hg)	76.8 ± 11.3 (69, 84)
Hypertension (systolic ≥ 140 or diastolic ≥ 90 or medicated)	19.7 (379)
Metabolic syndrome^c^	9.1 (152)

*Dietary intake*	
BCAA, % protein/d	18.1 ± 0.4 (17.9, 18.4)
Protein, g/d	81.8 ± 22.0 (66.9, 95.6)
Energy, kcal/d	1991 ± 534 (1611, 2339)
Carbohydrate, g/d	256 ± 76.2 (202, 304)
Saturated fat, g/d	26.4 ± 10.5 (18.8, 33.0)
Wholegrains, g/d	89.2 ± 78.3 (31.6, 129)
Alcohol, g/d	10.1 ± 13.9 (1.2, 13.5)
Energy intake: EER	86.4 ± 24.7 (68.7, 102)

a Values are mean ± SD (IQR) or percentages (frequencies), *n* = 1997. BCAA = branched chain amino acids; EER = estimated energy requirements; HDL-C = high density lipoprotein cholesterol; HOMA-IR = homeostasis model assessment of insulin resistance; hs-CRP = high-sensitivity C-reactive protein; TG = triglycerides.

b Subset analysis: hs-CRP = 1432; lipids = 1845; body fat = 1792; waist to height ratio = 1828; blood pressure = 1952.

c Metabolic syndrome was defined as the presence of three of the following risk factors, glucose ≥ 6.1 mmol/L, TG ≥ 1.7 mmol/L, HDL-C ≤ 1.3 mmol/L, waist circumference ≥ 88 cm or elevated blood pressure (systolic ≥ 130 and/or diastolic ≥ 85 and/or antihypertensive drug treatment), *n* = 1663.

**Table 2:** Adiposity and cardiometabolic markers by quintile of BCAA intake in females aged 18–76 y^a^.

	n =	Quintile 1	Quintile 2	Quintile 3	Quintile 4	Quintile 5	P-trend
BCAA intake (% protein)	1997	17.6 (0.2)	17.9 (0.1)	18.1 (0.1)	18.3 (0.1)	18.7 (0.2)	–
Weight (kg)	1997	65.5 (65.0, 66.1)	65.7 (65.2, 66.3)	65.9 (65.3, 66.4)	65.2 (64.7, 65.8)	64.8 (64.2, 65.5)	0.05
Body fat (% total body mass)	1792	33.8 (33.2, 34.4)	34.1 (33.6, 34.7)	34.5 (34.0, 35.1)	33.6 (33.1, 34.2)	34.6 (34.0, 35.2)	0.28
Waist to height ratio	1828	0.49 (0.48, 0.50)	0.49 (0.48, 0.49)	0.49 (0.48, 0.49)	0.48 (0.48, 0.49)	0.48 (0.48, 0.49)	0.04
HOMA-IR	1997	1.3 (1.2, 1.4)	1.3 (1.2, 1.4)	1.2 (1.2, 1.3)	1.2 (1.1, 1.3)	1.2 (1.2, 1.3)	0.02
Insulin (μU/mL)	1997	6.6 (6.2, 7.0)	6.5 (6.1, 6.9)	6.2 (5.8, 6.5)	6.1 (5.8, 6.5)	6.1 (5.8, 6.5)	0.03
Glucose (mmol/L)	1997	4.5 (4.4, 4.6)	4.5 (4.5, 4.6)	4.5 (4.4, 4.5)	4.5 (4.4, 4.5)	4.5 (4.4, 4.5)	0.13
hs-CRP (mg/L)	1432	1.7 (1.5, 1.9)	1.7 (1.5, 1.9)	1.7 (1.5, 1.9)	1.5 (1.4, 1.7)	1.4 (1.3, 1.6)	0.01
Lipids (*log*TG:HDL-C)	1845	0.08 (0.04, 0.11)	0.06 (0.02, 0.09)	0.07 (0.04, 0.11)	0.05 (0.02, 0.09)	0.06 (0.03, 0.10)	0.61
Systolic blood pressure (mm Hg)	1952	121 (120, 123)	121 (119, 122)	120 (118, 121)	119 (118, 121)	119 (117, 120)	0.01
Diastolic blood pressure (mm Hg)	1952	76.1 (75.0, 77.3)	76.7 (75.7, 77.8)	75.8 (74.7, 77.0)	75.7 (74.7, 76.7)	75.4 (74.3, 76.4)	0.16

a Values are adjusted geometric means (95% CI) except for intake values which are unadjusted means (SD) and lipid values which are adjusted means (95% CI), *n* = 1997. Means are adjusted for age, smoking, physical activity, BMI, HRT, menopausal status, use of diabetes or cholesterol lowering drugs, vitamin supplements, under-reporting, and intakes of energy, carbohydrate, saturated fat, wholegrains, alcohol and protein. Weight and waist to height ratio are not adjusted for BMI and blood pressure was additionally adjusted for use of anti-hypertensive medication. BCAA = branched chain amino acids; HDL-C = high density lipoprotein cholesterol; HOMA-IR = homeostasis model assessment of insulin resistance; hs-CRP = high-sensitivity C-reactive protein; TG = triglycerides.

**Table 3:** Within-pair associations between BCAA intake, adiposity and cardiometabolic markers in female twin pairs aged 18–76 y, stratified by zygosity^a^.

	All twins			Monozygotic twins			Dizygotic twins		
	n =	β_i_ % (95%CI)	*P* =	n =	β_i_ % (95%CI)	*P* =	n =	β_i_ % (95%CI)	*P* =
Body fat (% total body mass)	857	− 0.2 (− 0.6, 0.1)	0.54	185	− 0.5 (− 1.1, 0.8)	0.38	672	− 0.2 (− 0.6, 0.3)	0.71
Weight (kg)	960	− 0.2 (− 0.8, 0.4)	0.21	222	− 0.4 (− 1.3, 0.5)	0.09	738	− 0.1 (− 0.9, 0.6)	0.41
Waist to height ratio	879	− 0.2 (− 0.6, 0.1)	0.21	189	− 0.1 (− 0.8, 0.6)	0.76	690	− 0.3 (− 0.8, 0.2)	0.22
HOMA-IR	960	− 2.1 (− 3.9, − 0.2)	0.03	222	− 5.0 (− 8.1, − 1.9)	< 0.01	738	− 0.9 (− 3.1, 1.4)	0.45
Insulin (μU/mL)	960	− 2.0 (− 3.8, − 0.2)	0.03	222	− 4.8 (− 7.7, − 1.8)	< 0.01	738	− 0.9 (− 3.0, 1.2)	0.40
Glucose (mmol/L)	960	0.0 (− 0.4, 0.3)	0.78	222	− 0.2 (− 0.9, 0.5)	0.58	738	0.0 (− 0.4, 0.4)	0.89
hs-CRP (mg/L)	626	− 7.1 (− 11.7, − 2.3)	< 0.01	181	− 9.7 (− 17.7, − 1)	0.03	445	− 6.6 (− 12.1, − 0.7)	0.03
Lipids (*log*TG:HDL-C)	870	− 0.8 (− 2.2, 0.6)	0.34	184	− 0.9 (− 3.1, 1.3)	0.42	686	− 0.9 (− 2.5, 0.8)	0.32
Systolic blood pressure (mm Hg)	930	− 0.3 (− 0.8, 0.2)	0.19	207	− 0.9 (− 1.9, 0.1)	0.08	723	− 0.2 (− 0.7, 0.3)	0.48
Diastolic blood pressure (mm Hg)	930	− 0.1 (− 0.7, 0.4)	0.67	207	− 1.2 (− 2.3, − 0.1)	0.04	723	0.1 (− 0.6, 0.7)	0.83

a Values are the geometric means (95% CI) with the exception of lipids values which are means (95% CI), expressed as a percentage calculated from the β-coefficient given a one-quintile increase in BCAA intake with the twin-pair average held fixed, *n* = 1920. Values are adjusted for age, smoking, physical activity, BMI, HRT, menopausal status, use of diabetes or cholesterol lowering drugs, vitamin supplements, under-reporting, and intakes of energy, carbohydrate, saturated fat, wholegrains, alcohol and protein. Weight and waist to height ratio are not adjusted for BMI and blood pressure was additionally adjusted for use of anti-hypertensive medication. HDL-C = high density lipoprotein cholesterol; HOMA-IR = homeostasis model assessment of insulin resistance; hs-CRP = high-sensitivity C-reactive protein; TG = triglycerides.

## Appendix A Supplementary data

Supplementary tables Image 1
