# Supplementary material for: Associations between branched chain amino acid intake and biomarkers of adiposity and cardiometabolic health independent of genetic factors: A twin study
Source: Int J Cardiol. 2016 Nov 15;223:992–8. doi: 10.1016/j.ijcard.2016.08.307 (PMC5074005; doi:10.1016/j.ijcard.2016.08.307)
Supplement: Supplementary file 1 — Supplementary tables [file mmc1.docx]

**Supplemental table 1: Associations between BCAA intake and metabolites of amino acid metabolism^1^**

| **Metabolite** | **Β (95% CI)** | **P value** |
| --- | --- | --- |
| 2-aminobutyrate | 0.01 (-0.07,0.1) | 7.52E-01 |
| 2-aminooctanoic acid | -0.05 (-0.13,0.02) | 1.79E-01 |
| 2-hydroxybutyrate (AHB) | -0.04 (-0.12,0.05) | 3.94E-01 |
| 2-hydroxyisobutyrate | -0.09 (-0.18,0) | 6.23E-02 |
| 2-methylbutyroylcarnitine | -0.02 (-0.1,0.06) | 6.32E-01 |
| 3-(3-hydroxyphenyl)propionate | -0.05 (-0.24,0.13) | 5.79E-01 |
| 3-(4-hydroxyphenyl)lactate | -0.01 (-0.09,0.07) | 7.70E-01 |
| 3,4-dihydroxybutyrate | -0.06 (-0.14,0.02) | 1.47E-01 |
| 3-indoxyl sulfate | -0.05 (-0.13,0.03) | 1.89E-01 |
| 3-methoxytyrosine | -0.06 (-0.15,0.04) | 2.43E-01 |
| 3-methyl-2-oxobutyrate | -0.01 (-0.09,0.07) | 7.63E-01 |
| 3-methyl-2-oxovalerate | -0.04 (-0.12,0.05) | 3.87E-01 |
| 3-methylhistidine | 0.01 (-0.08,0.1) | 8.66E-01 |
| 3-phenylpropionate (hydrocinnamate) | 0.09 (0,0.17) | 4.19E-02 |
| 4-acetamidobutanoate | -0.09 (-0.17,-0.02) | 1.93E-02 |
| 4-methyl-2-oxopentanoate | -0.02 (-0.1,0.06) | 6.62E-01 |
| 5-oxoproline | -0.001 (-0.08,0.08) | 9.86E-01 |
| Alanine | -0.02 (-0.09,0.06) | 6.69E-01 |
| **Alpha-hydroxyisovalerate** | **-0.15 (-0.23,-0.07)** | **2.99E-04** |
| Arginine | -0.02 (-0.1,0.06) | 6.39E-01 |
| Asparagine | 0.04 (-0.04,0.12) | 3.51E-01 |
| Aspartate | -0.07 (-0.15,0.01) | 8.30E-02 |
| Beta-hydroxyisovalerate | -0.004 (-0.09,0.08) | 9.25E-01 |
| Betaine | -0.05 (-0.14,0.03) | 2.10E-01 |
| C-glycosyltryptophan | -0.02 (-0.09,0.05) | 5.39E-01 |
| Citrulline | 0.003 (-0.07,0.08) | 9.32E-01 |
| Creatine | -0.05 (-0.14,0.03) | 2.26E-01 |
| Creatinine | -0.02 (-0.1,0.06) | 6.36E-01 |
| Cysteine | -0.08 (-0.16,0) | 4.76E-02 |
| Dimethylarginine (SDMA + ADMA) | 0.06 (-0.02,0.15) | 1.32E-01 |
| Glutamate | 0.03 (-0.05,0.1) | 4.99E-01 |
| Glutamine | 0.03 (-0.05,0.11) | 3.96E-01 |
| Glutaroyl carnitine | -0.03 (-0.12,0.05) | 4.24E-01 |
| Glycine | 0.04 (-0.04,0.12) | 3.43E-01 |
| Histidine | 0.002 (-0.08,0.08) | 9.56E-01 |
| Homocitrulline | -0.09 (-0.2,0.03) | 1.50E-01 |
| Hydroxyisovaleroyl carnitine | 0.05 (-0.06,0.15) | 3.82E-01 |
| Hydroxytryptophan | -0.01 (-0.08,0.07) | 8.86E-01 |
| Indoleacetate | -0.09 (-0.18,-0.01) | 2.81E-02 |
| Indolelactate | -0.03 (-0.12,0.05) | 4.67E-01 |
| Indolepropionate | -0.04 (-0.13,0.04) | 2.94E-01 |
| Isobutyrylcarnitine | 0.04 (-0.04,0.12) | 2.75E-01 |
| Isoleucine | -0.04 (-0.11,0.04) | 3.48E-01 |
| Isovalerylcarnitine | -0.01 (-0.09,0.07) | 8.10E-01 |
| Kynurenine | 0.03 (-0.05,0.1) | 5.06E-01 |
| Leucine | 0.005 (-0.07,0.08) | 9.05E-01 |
| Levulinate (4-oxovalerate) | -0.01 (-0.09,0.06) | 7.22E-01 |
| Lysine | 0.06 (-0.02,0.14) | 1.30E-01 |
| Methionine | 0.08 (0,0.17) | 4.01E-02 |
| N-[3-(2-Oxopyrrolidin-1-yl)propyl]acetamide | -0.11 (-0.19,-0.03) | 7.71E-03 |
| N-acetylalanine | -0.05 (-0.13,0.02) | 1.71E-01 |
| N-acetylglycine | -0.02 (-0.11,0.06) | 5.48E-01 |
| N-acetylornithine | -0.04 (-0.12,0.05) | 3.91E-01 |
| N-acetylthreonine | 0.01 (-0.07,0.09) | 8.58E-01 |
| Ornithine | -0.03 (-0.11,0.04) | 3.89E-01 |
| O-sulfo-L-tyrosine | -0.12 (-0.21,-0.04) | 4.55E-03 |
| P-cresol sulfate | 0.02 (-0.06,0.1) | 6.79E-01 |
| Phenol sulfate | -0.11 (-0.19,-0.03) | 1.01E-02 |
| Phenylacetate | 0.05 (-0.05,0.14) | 3.34E-01 |
| Phenylacetylglutamine | 0.02 (-0.06,0.1) | 6.80E-01 |
| Phenylalanine | 0.11 (0.03,0.19) | 8.46E-03 |
| Phenyllactate (PLA) | -0.03 (-0.12,0.06) | 5.02E-01 |
| Pipecolate | -0.12 (-0.2,-0.03) | 5.83E-03 |
| Proline | -0.03 (-0.11,0.05) | 4.87E-01 |
| Pyroglutamine | 0.002 (-0.07,0.08) | 9.50E-01 |
| Serine | 0.003 (-0.07,0.08) | 9.33E-01 |
| Serotonin (5HT) | -0.03 (-0.11,0.06) | 5.47E-01 |
| Threonine | -0.05 (-0.14,0.05) | 3.42E-01 |
| **Trans-4-hydroxyproline** | **-0.2 (-0.28,-0.12)** | **1.48E-06** |
| Tryptophan | 0.07 (-0.01,0.15) | 8.62E-02 |
| Tryptophan betaine | -0.13 (-0.22,-0.04) | 5.21E-03 |
| Tyrosine | 0.08 (0,0.16) | 6.40E-02 |
| Urea | 0.004 (-0.07,0.08) | 9.25E-01 |
| Valine | 0.01 (-0.06,0.09) | 7.65E-01 |

^1^Values are the beta-coefficeints (95%CI), n=3299. Values are adjusted for age, BMI, batch effects, family relatedness, smoking and energy intake. Significant values after Bonferroni correction are shown in bold.

**Supplemental table 2: Prevalence ratios for cardio-metabolic risk factors by quintile of BCAA intake in females aged 18-76 y**^1^

|  | **n=** | **Quintile 1** | **Quintile 2** | **Quintile 3** | **Quintile 4** | **Quintile 5** | **P-trend** |
| --- | --- | --- | --- | --- | --- | --- | --- |
| Insulin resistance | 1997 | Reference | 0.9 (0.6, 1.3) | 0.8 (0.6, 1.1) | 0.7 (0.4, 1.0) | 0.7 (0.5, 1.0) | 0.03 |
| Inflammation | 1432 | Reference | 1.1 (0.9, 1.4) | 1.1 (0.9, 1.4) | 0.9 (0.7, 1.1) | 0.8 (0.6, 1.0) | 0.02 |
| Dyslipidemia | 1845 | Reference | 0.6 (0.3, 1.0) | 0.9 (0.5, 1.4) | 0.7 (0.5, 1.2) | 0.8 (0.5, 1.3) | 0.68 |
| Overweight | 1997 | Reference | 0.6 (0.4, 0.9) | 1.0 (0.7, 1.4) | 0.7 (0.5, 1.0) | 0.6 (0.4, 0.9) | 0.03 |
| Abdominal obesity | 1828 | Reference | 1.0 (0.8, 1.2) | 1.0 (0.8, 1.1) | 0.9 (0.8, 1.1) | 0.9 (0.7, 1.0) | 0.09 |
| Hypertension | 1952 | Reference | 1.0 (0.8, 1.3) | 1.0 (0.8, 1.3) | 0.9 (0.7, 1.1) | 0.8 (0.6, 1.0) | 0.02 |
| Metabolic syndrome | 1663 | Reference | 0.7 (0.4, 1.2) | 0.9 (0.6, 1.5) | 0.8 (0.5, 1.3) | 0.7 (0.4, 1.2) | 0.37 |

^1^Values are adjusted prevalence ratios (95% CI), n=1997. Prevalence ratios are adjusted for age, smoking, physical activity, BMI, HRT, menopausal status, use of diabetes or cholesterol lowering drugs, vitamin supplements, under-reporting, and intakes of energy, carbohydrate, saturated fat, wholegrains, alcohol and protein. Overweight and abdominal obesity are not adjusted for BMI. Insulin resistance = HOMA-IR ≥ 2.5; systemic inflammation = hs-CRP ≥3 mg/L, dyslipidemia = HDL-C ≤ 1.3 mmol/L and TG ≥ 1.7 mmol/L or use of cholesterol lowering drugs; obesity = BMI ≥ 25 kg/m^2^; abdominal obesity = waist to height ratio ≥0.5; hypertension = systolic blood pressure ≥140 mm Hg and/or diastolic blood pressure ≥ 90 mm Hg and/or anti-hypertensive drug treatment; metabolic syndrome = three of the following risk factors, glucose ≥6.1 mmol/L, TG ≥1.7 mmol/L, HDL-C ≤1.3 mmol/L, waist circumference ≥88 cm or elevated blood pressure (systolic ≥130 and/or diastolic ≥85 and/or antihypertensive drug treatment).
